# Geographically Targeted Interventions versus Mass Drug Administration to Control *Taenia solium* Cysticercosis, Peru

**DOI:** 10.3201/eid2709.203349

**Published:** 2021-09

**Authors:** Seth E. O’Neal, Ian W. Pray, Percy Vilchez, Ricardo Gamboa, Claudio Muro, Luz Maria Moyano, Viterbo Ayvar, Cesar M. Gavidia, Robert H. Gilman, Armando E. Gonzalez, Hector H. Garcia, for the Cysticercosis Working Group in Peru

**Affiliations:** Oregon Health & Science University–Portland State University School of Public Health, Portland, Oregon, USA (S.E. O’Neal, I.W. Pray);; Universidad Peruana Cayetano Heredia, Lima, Peru (S.E. O’Neal, P. Vilchez, R. Gamboa, C. Muro, L.M. Moyano, H.H. Garcia);; Universidad Nacional Mayor de San Marcos, Lima (V. Ayvar, C.M. Gavidia, A.E. Gonzalez);; Johns Hopkins University, Baltimore, Maryland, USA. (R.H. Gilman)

**Keywords:** Neglected tropical diseases, pork tapeworm, zoonoses, One Health, taeniasis, neurocysticercosis, public health intervention, epidemiology, One Health, food safety, parasites, enteric infections, Peru

## Abstract

Optimal control strategies for *Taenia solium* taeniasis and cysticercosis have not been determined. We conducted a 2-year cluster randomized trial in Peru by assigning 23 villages to 1 of 3 geographically targeted intervention approaches. For ring screening (RS), participants living near pigs with cysticercosis were screened for taeniasis; identified cases were treated with niclosamide. In ring treatment (RT), participants living near pigs with cysticercosis received presumptive treatment with niclosamide. In mass treatment (MT), participants received niclosamide treatment every 6 months regardless of location. In each approach, half the villages received targeted or mass oxfendazole for pigs (6 total study arms). We noted significant reductions in seroincidence among pigs in all approaches (67.1% decrease in RS, 69.3% in RT, 64.7% in MT; p<0.001), despite a smaller proportion of population treated by targeted approaches (RS 1.4%, RT 19.3%, MT 88.5%). Our findings suggest multiple approaches can achieve rapid control of *T. solium* transmission.

*Taenia solium* is a zoonotic cestode that infects both humans and pigs (Figure 1). Human brain infection, neurocysticercosis, is a major cause of preventable epilepsy across much of Asia, Africa, and Latin America (*1*); ≈1.35 million persons in Latin America and ≈3 million persons in Africa have epilepsy thought to be secondary to neurocysticercosis (*2*,*3*). Porcine cysticercosis is a food safety hazard and source of economic harm in rural regions where the parasite is endemic and of increasing public health concern because of the rapidly growing global demand for pork (*4*). The United Nations Food and Agriculture Organization (https://www.fao.org) ranks *T. solium* as a major foodborne parasite on the basis of global likelihood of exposure and potential severity of infection (*5*). In the United States, hospitalizations for cysticercosis exceed those for all other neglected tropical diseases combined (*6*).

**Figure 1:**
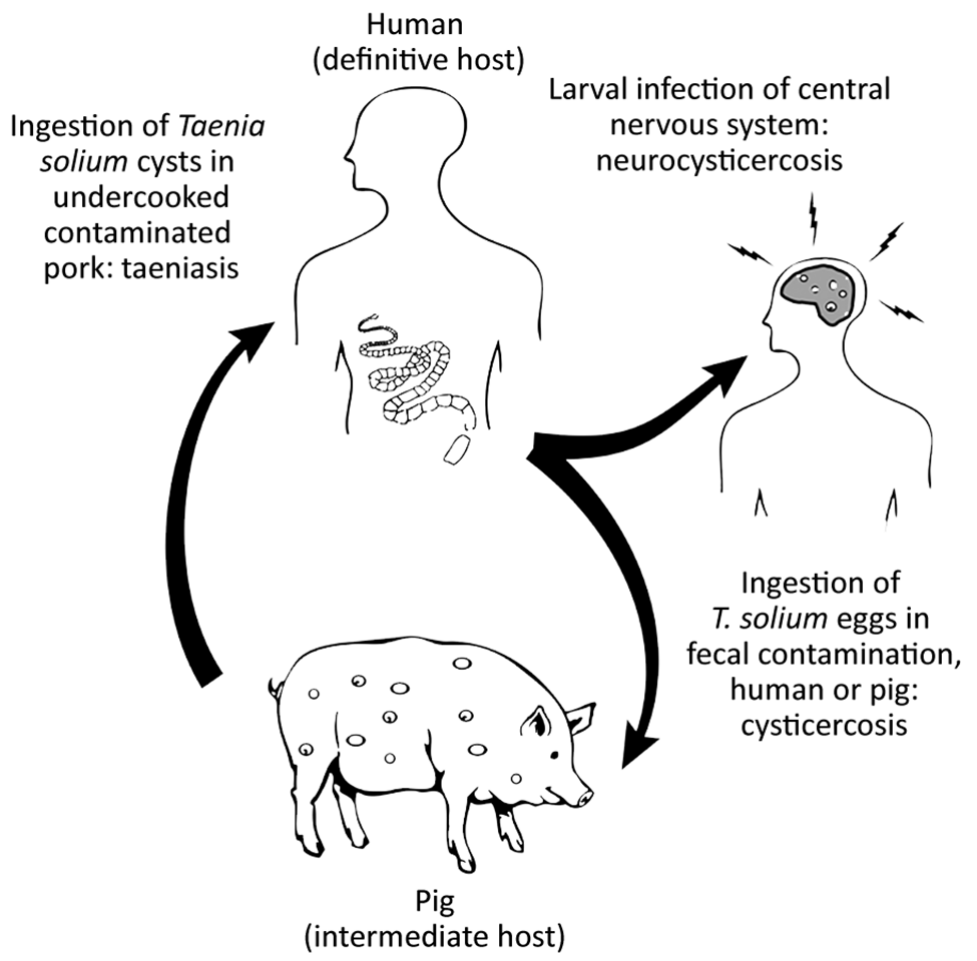
Lifecycle of the *Taenia solium* tapeworm in humans and pigs.

One of the targets of the 2011 World Health Organization roadmap to overcome neglected tropical diseases is to validate *T. solium* control and elimination strategies and scale up taeniasis and cysticercosis interventions (*7*). Several different interventions to control transmission have been attempted, including mass treatment for taeniasis (*8*–*10*), combined mass treatment for taeniasis and porcine cysticercosis (*8*,*11*), targeted screening and treatment for taeniasis (*12*), pig vaccination (*13*), improvements in sanitation (*14*), and various education interventions (*15*,*16*). However, most studies have been limited by small scale or inconsistent monitoring, making conclusions regarding effectiveness and generalizability uncertain. No clear indication has yet determined which control strategies will be feasible and effective. 

We previously completed a pilot study in Peru to evaluate a targeted ring approach to control transmis-sion of *T. solium*, which exhibits spatial clustering (*12*). The premise of this approach is that selective treatment for taeniasis among high-risk subgroups within villages might reduce transmission and limit the number of persons treated (*17*). We offered screening and treatment for taeniasis within groups of households located near pigs that had visible cyst infection during periodic surveillance. We noted a 50% relative reduction in transmission within the intervention village compared with the negative control village (*12*), but a larger randomized trial could help validate this approach. We conducted a follow-up study to compare effectiveness of 2 ring approaches and mass treatment, and to explore whether including treatment for cysticercosis in pigs provided additional control benefits.

## Methods

### Study Design

**Figure 2:**
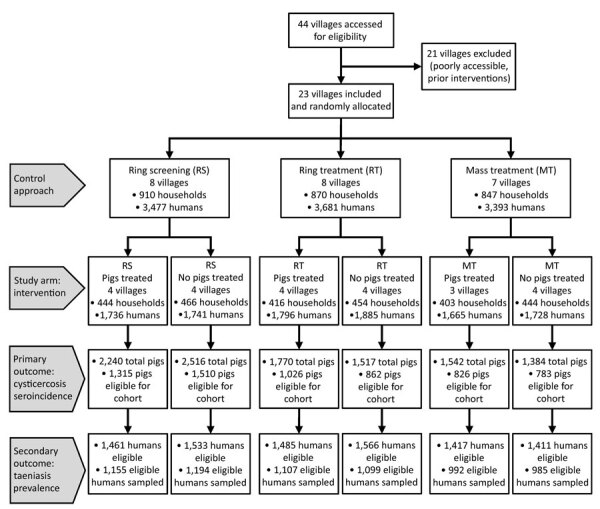
Flowchart of participating villages, humans, and pigs in a study of *Taenia solium* intervention strategies, Peru. Humans were treated with niclosamide, pigs (when treated) with oxfendazole. MT, mass treatment; RS, ring screening; RT, ring treatment.

**Figure 3:**
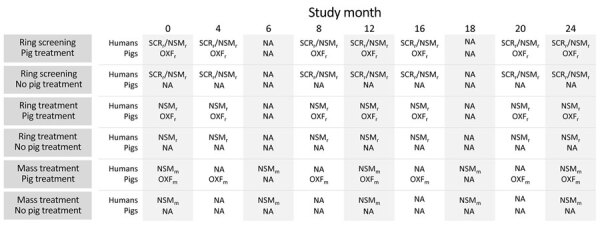
Timeline showing interventions in humans and pigs during a study of *Taenia solium* tapeworms, Peru. NSM_m_, presumptive treatment with niclosamide for humans; NA, not applicable; NSM_r_, presumptive treatment with niclosamide for humans only in rings; OXF_m_, presumptive treatment with oxfendazole for pigs; OXF_r_, presumptive treatment with oxfendazole for pigs only in rings; SCR_r_/NSM_r_, stool screening and treatment with niclosamide for humans with diagnosed taeniasis only in rings.

We conducted a community cluster randomized trial with a 3 × 2 factorial design. We randomly assigned 23 villages (total population 10,551) to 1 of 6 study arms (Figures 2, 3). Each study arm corresponded to a unique intervention comprised of an approach to deliver the antiparasitic drug niclosamide, for human taeniasis. The 6 study arms were ring screening (RS), ring treatment (RT), or mass treatment (MT), with or without antiparasitic drug treatment with oxfendazole for cysticercosis in pigs.

### Outcome Measures

The primary outcome was seroincidence of *T. solium* antibodies in all pigs born into the villages during the 2-year study period. The secondary outcome was prevalence of human taeniasis at study end.

### Study Sites and Participants

We conducted the study during 2015–2017 in Piura, Peru, an agricultural region where *T. solium* is endemic. Outdoor defecation is common among humans and pigs roam free, a combination that places pigs at high risk for cysticercosis. Villages of 50–500 residents were eligible to participate; 43 villages met this criterium. We selected 23 villages because they were accessible year-round and had no history of control interventions for taeniasis or cysticercosis (Appendix). All residents >2 years of age were eligible to participate. The study was approved by the institutional review boards for human (approval no. IRB00010117) and animal (approval no. IP00000617) research at Oregon Health & Science University–Portland State University, Portland, Oregon, USA, and Universidad Peruana Cayetano Heredia, Lima, Peru (approval no. 62206).

### Baseline Census

We conducted a door-to-door census in villages to collect information on demographics, household sanitation, and pig husbandry. We used global positioning system receivers (Trimble, https://www.trimble.com) with post-processed differential correction to collect coordinates of each house, then created a georeferenced map of each village by using ArcMAP10 (Environmental Systems Research Institute, https://www.esri.com) and a 100-m buffer around each household to define extent of future intervention rings (*12*).

### Randomization

We randomly assigned the 23 villages to 1 of 6 study arms, repeating the allocation sequence until the human population in all 6 arms was approximately equal, within 10% of the study population divided by 6 (Appendix). We considered no other factors in assigning villages.

### Interventions

In the MT approach, we returned to each village every 6 months and went door-to-door to offer residents >2 years of age presumptive treatment for taeniasis with a single oral dose of niclosamide. Persons who weighed 11–34 kg received 1 g niclosamide, persons who weighed 35–50 kg received 1.5 g, and persons weighing >50 kg received 2 g. We chose the 6-month interval to be consistent with the frequency of mass drug administration (MDA) recommended by the World Health Organization for other helminths (*18*). During each treatment cycle, we returned to households >1 additional time to locate persons who were absent when treatment initially was offered. We did not collect stool samples in the MT approach.

In the RT approach, we returned to each village every 4 months to perform active surveillance for heavily infected pigs. Surveillance included visiting all households, catching all pigs, and examining pigs’ tongues for visible or palpable cysts (*19*). We returned to households >1 additional time if any pigs evaded capture or were otherwise unaccounted for during the first visit. When we identified a pig with cysticercosis of the tongue, we opened a treatment ring comprising all households within a 100-m radius of the house where the tongue-positive pig was raised. We offered all persons >2 years of age living within the treatment ring the standard oral niclosamide dose for taeniasis and a second oral dose 15 days later. We used 2 doses because single-dose treatment failure is common in this region (*20*). We did not collect stool samples in the RT approach. We offered to purchase all cysticercosis tongue-positive pigs and remove these pigs from the village; if the owner did not agree to sell the pig, we treated it with a single 30 mg/kg dose of oxfendazole, as recommended (*21*).

In the RS approach, we conducted active surveillance for heavily infected pigs as described in the RT approach. When we identified a cysticercosis tongue-positive pig, we requested a single stool sample from each person >2 years of age living in a 100-m radius of the house where the infected pig was raised. We tested stool samples for *Taenia* sp. eggs or antigens and only offered niclosamide single-dose treatment to persons with diagnosed taeniasis. We collected a follow-up stool sample from taeniasis-positive persons 30 days after treatment to verify cure and retreated persistent infections. We purchased cysticercosis tongue-positive pigs or treated with oxfendazole as described in the RT approach.

In half of the villages in each approach, we treated pigs >6 weeks of age for cysticercosis by using a single oral dose of 30 mg/kg of oxfendazole. In the MT approach, we treated all pigs in the village at 4-month intervals. In the RT and RS approaches, we treated only pigs owned by households within a 100-m ring of a cysticercosis tongue-positive pig. Owners were instructed not to slaughter pigs within 21 days after treatment so that the drug would clear from tissues before human consumption (*22*).

### Measurement of Primary Outcome

We conducted serosurveys of the pig population every 4 months in all 23 villages to determine seroincidence of antibodies against cysticercosis. During each serosurvey, veterinary staff visited each household, captured all pigs >6 weeks of age, collected a 5-mL blood sample, placed an ear tag with a unique identifier on new pigs, and updated the pig census. Pigs 6 weeks–4 months of age when first captured were placed into a cohort for incidence calculations. We followed the serologic antibody response of every pig in this cohort through subsequent serosurveys until an antibody response developed in the pig (primary outcome) or the pig was lost to follow-up because it died, was sold, evaded capture or other reasons. The seroincidence reported at each sampling point reflects the risk for exposure during the preceding 4-month interval.

### Measurement of Secondary Outcome

At study end (month 24), we determined the prevalence of taeniasis in all 23 villages. We offered presumptive treatment with niclosamide to all residents >2 years of age, requested collection of the first posttreatment stool in a 500-mL plastic container, and collected stool samples for testing within 24 hours.

### Laboratory Procedures

We centrifuged pig blood samples to separate serum, froze serum at −20°C, and later processed it for antibodies against porcine cysticercosis by using lentil-lectin glycoprotein enzyme-linked immunoelectrotransfer blot, as previously described (*23*), except we considered results positive when a reaction occurred to any of the 6 glycoprotein (GP) antigens, GP39/42, GP24, GP21, GP18, GP14, or GP13. We excluded the GP50 antigen because recent studies have shown this band cross-reacts with *T. hydatigena*, a cestode that infects pigs and is coendemic in the region (*24*). We examined human stool samples macroscopically for *Taenia* sp. scoleces or proglottids, then prepared fecal aliquots in 5% formol-phosphate buffered saline (Appendix). We used ELISA to detect *Taenia* sp. coproantigens in aliquots, as previously described (*25*).

### Statistical Analysis

We analyzed data in Stata SE14.2 (StataCorp LLC, https://www.stata.com). To evaluate pig seroincidence, we used binomial family generalized estimating equations with log-link and exchangeable correlation structure. We aggregated individual pig-level data into panel format to reflect the hierarchical structure of study arm, village, house, and intervention round, then further stratified by age category (0–4, 5–8, 9–12, and >13 months). We set village as the panel variable and used robust sandwich-type errors to account for intrahousehold clustering. We used quasilikelihood information criteria to select variables for the final model and retained variables that decreased criteria value relative to the saturated model. The final model variables were study arm, intervention round, baseline village seroprevalence, presence or absence of household latrine and pig corral, pig age, and oxfendazole treatment for pigs. We included 2- and 3-way interactions for study arm × intervention round × oxfendazole to evaluate any additional effect of including pig treatment in interventions. We considered p<0.05 statistically significant. We then used margins command to estimate predicted probabilities (cumulative seroincidence) and absolute differences within each study arm over time and between study arms. For the taeniasis prevalence, we used a separate binomial family generalized estimating equation with log-link that included participant age, number of pigs in village, and baseline village seroprevalence.

## Results

### Village Assignment and Characteristics

The total population of all 23 villages was 10,551; 10,094 (95.7%) persons were >2 years and eligible to participate (Table 1; Figure 2). Compared with other study approaches, the MT approach had more latrines, fewer pigs, and a lower baseline seroprevalence.

**Table 1:** Village and household characteristics at baseline in each arm of a study on control of *Taenia solium* cysticercosis, Peru*

Characteristics	Ring screening			Ring treatment			Mass treatment	
	Pig treatment	No pig treatment		Pig treatment	No pig treatment		Pig treatment	No pig treatment
No. villages	4	4		4	4		3	4
Human residents	1,736 (16.5)	1,741 (16.5)		1,796 (17.0)	1,885 (17.9)		1,665 (15.8)	1,728 (16.4)
Residents >2 y of age	1,662 (16.5)	1,666 (16.5)		1,736 (17.2)	1,789 (17.7)		1,594 (15.8)	1,647 (16.3)
No. pigs at baseline	457	556		349	395		369	305
Seropositive pigs	194 (42.5)	224 (40.3)		148 (42.4)	190 (48.1)		141 (38.2)	96 (31.5)
Households	444 (16.9)	466 (17.7)		416 (15.8)	454 (17.3)		403 (15.3)	444 (16.9)
Latrine	249 (58.1)	346 (74.3)		249 (59.9)	311 (68.5)		330 (81.9)	341 (76.8)
Treated water source	394 (88.7)	427 (91.6)		291 (70.0)	352 (77.5)		357 (88.6)	350 (78.8)
Raise pigs	230 (51.8)	251 (53.9)		217 (52.2)	241 (53.1)		178 (44.2)	253 (57.0)
Corral for pigs	146 (63.5)	132 (52.6)		82 (37.8)	118 (49.0)		114 (64.0)	107 (42.3)

*Values are no. (%), except as indicated.

### Interventions Applied

In MT, we conducted 5 rounds of MDA with niclosamide to an age-eligible population of 3,329 persons (Table 2); 1,240 (37.3%) participants received all 5 rounds, 583 (17.5%) in 4 rounds, 411 (12.4%) in 3 rounds, 354 (10.6%) in 2 rounds, 359 (10.8%) in 1 round, and 382 (11.5%) were not treated. We treated 88.5% (2,641/3,329) of the age-eligible population with >1 dose.

**Table 2:** Summary of participation in mass treatment intervention in a study on control of *Taenia solium* cysticercosis, Peru*

Characteristics	Study month					Total
	0	6	12	18	24	
No. eligible households	799	794	816	804	815	4,028
No. eligible participants	2,994	2,973	3,021	2,956	2,998	14,942
Not treated, no. (%)	709 (23.7)	743 (25.0)	819 (27.1)	755 (25.5)	730 (24.4)	3,756 (25.1)
Took >1 dose of NSM, no. (%)	2,285 (76.3)	2,230 (75.0)	2,202 (72.9)	2,201 (74.5)	2,268 (75.7)	11,186 (74.9)

*Distinct households and participants might be counted more than once in the totals column. NSM, niclosamide.

In RT, we conducted 7 rounds of surveillance and examined tongues of 5,764 pigs (Table 3). We identified 37 tongue-positive pigs, resulting in 37 distinct screening rings. We purchased and removed 20 (54.1%) pigs; 17 (45.9%) pigs were treated with oxfendazole and remained with their owners. A total of 803/3,525 (22.8%) age-eligible persons in 183/870 (21.0%) households were included in a treatment ring in >1 surveillance round; 538 (67.0%) persons were offered niclosamide in 1 round, 202 (25.2%) in 2 rounds, 48 (6.0%) in 3 rounds, and 15 (1.9%) in 4 rounds. We treated 19.3% (680/3,525) of the overall age-eligible population with >1 dose.

**Table 3:** Summary of surveillance and participation in ring treatment intervention in a study of *Taenia solium* cysticercosis, Peru*

Characteristics	Study month							Total
	0	4	8	12	16	20	24	
No. pigs examined	748	625	783	751	937	931	989	5,764
Tongue-positive pigs, no. (%)	7 (0.9)	6 (1.0)	6 (0.8)	4 (0.5)	2 (0.2)	9 (1.0)	4 (0.4)	38 (0.7)
No. screening rings	7	6	7	3	2	9	3	37
No. eligible households	43	39	58	15	13	71	10	249
No. eligible participants	193	187	261	72	66	338	36	1,153
Not treated, no. (%)	14 (7.3)	35 (18.7)	32 (12.3)	10 (13.9)	14 (21.1)	56 (16.6)	13 (36.1)	174 (15.1)
Took 1 dose of NSM, no. (%)	23 (11.9)	36 (19.3)	31 (11.9)	4 (5.6)	4 (6.1)	67 (19.8)	2 (5.6)	167 (14.5)
Took 2 doses of NSM, no. (%)	156 (80.8)	116 (62.0)	198 (75.9)	58 (80.6)	48 (72.7)	215 (63.6)	21 (58.3)	812 (70.4)

*Distinct households, participants, and pigs might be counted more than once in the totals column. NSM, niclosamide.

In RS, we conducted 7 rounds of surveillance and examined tongues of 7,885 pigs (Table 4). We identified 74 tongue-positive pigs, resulting in 65 distinct screening rings, but 9 rings completely overlapped with others. We purchased and removed 57 (77.0%) pigs, 15 (20.3%) were treated and remained, and 2 (2.7%) were reported slaughtered and buried by the owner. A total of 1,475/3,328 (44.3%) age-eligible persons in 397/910 (43.6%) households were included in a screening ring in >1 surveillance round; 972 (65.9%) were included in 1 round, 455 (31.8%) in 2 rounds, and 48 (3.3%) in 3 rounds. We collected >1 stool sample from 1,231/1,475 (83.5%) participants; 51 (4.1%) persons tested positive. We screened 37.0% (1,231/3,328) of the overall age-eligible population and treated 1.4% (46) with niclosamide.

**Table 4:** Summary of surveillance and participation in ring screening intervention in a study of *Taenia solium* cysticercosis, Peru*

Characteristics	Study month							Total*
	0	4	8	12	16	20	24	
No. pigs examined	1,015	875	1,010	1,075	1,174	1,424	1,312	7,885
Tongue-positive pigs, no. (%)	23 (2.3)	3 (0.3)	0 (0)	12 (1.1)	17 (1.5)	5 (0.4)	14 (1.1)	74 (1.0)
No. screening rings	21	3	0	9	15	5	12	65
No. eligible households	170	24	0	53	150	25	124	546
No. eligible participants	625	90	0	220	532	107	452	2026
Provided stool (%)	548 (87.7)	73 (81.1)	0	185 (84.1)	422 (79.3)	83 (77.6)	352 (77.9)	1,663 (82.1)
Suspect taeniasis (%)	24 (4.4)	2 (2.7)	NA	5 (2.7)	18 (4.3)	0 (0)	12 (3.4)	61 (3.7)
Accepted NSM (%)	22 (91.7)	2 (100)	NA	5 (100)	15 (83.3)	NA	12 (100)	56 (91.8)

*Distinct households, participants, and pigs might be counted more than once in the totals column. NA, not applicable; NSM, niclosamide.

The primary reasons eligible persons did not receive niclosamide in all study arms included not being in the village at the time of intervention and participant refusal. The main reasons eligible pigs did not receive oxfendazole were pregnancy and inability to capture the animal.

### Porcine Seroincidence

We captured 10,969 distinct pigs over the 24-month study, of which 6,322 (57.6%) were eligible for seroincidence monitoring; 2,825 (44.7%) in RS, 1,888 (29.9%) in RT, and 1,609 (25.5%) in MT. We collected 11,165 blood samples from the eligible cohort. Some pigs were sampled during >1 round; 3,132 (49.5%) had 1 sample, 1,938 (30.7%) had 2 samples, and 1,252 (19.8%) had >3 samples.

**Figure 4:**
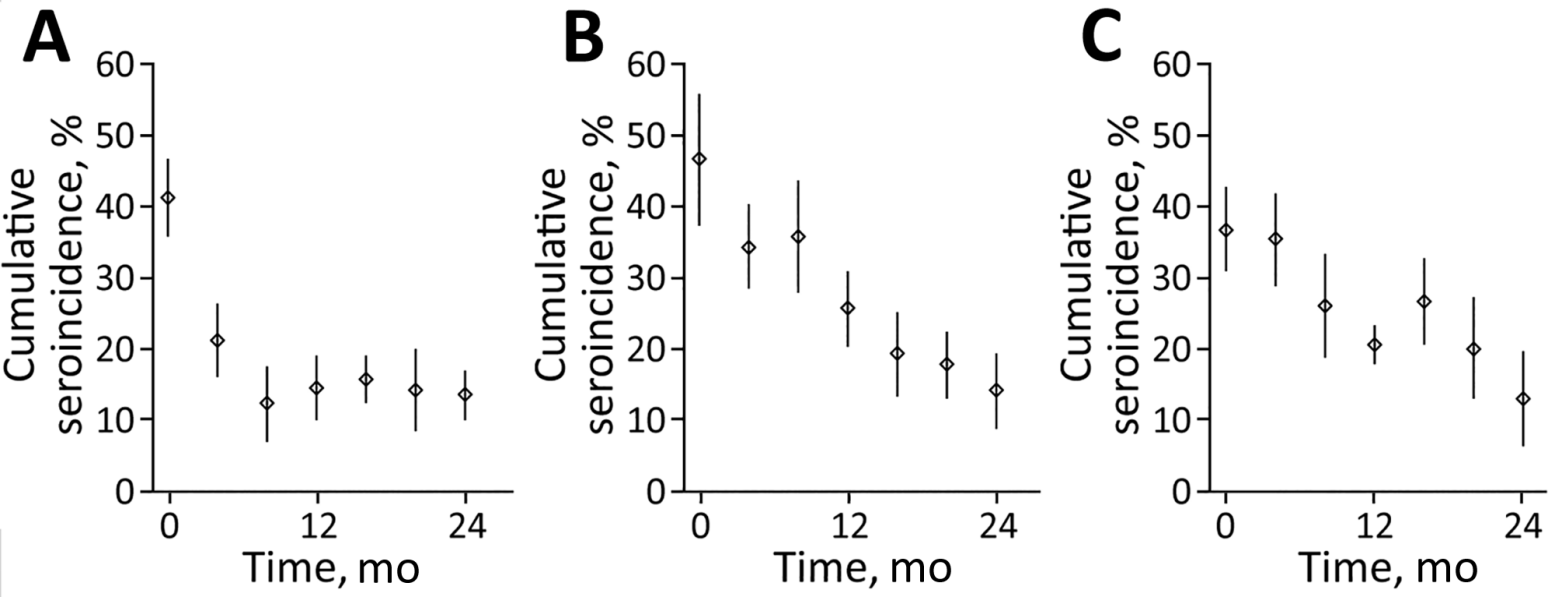
Cumulative *Taenia solium* seroincidence among pigs by study approach over time, Peru. A) Ring screening; B) ring treatment; C) mass treatment. In ring screening, human participants living near pigs with cysticercosis were screened for taeniasis using stool coproantigen; identified cases were treated with niclosamide. In ring treatment, human participants living near pigs with cysticercosis received presumptive treatment with niclosamide. In mass treatment, human participants received treatment with niclosamide every 6 months regardless of location. Diamonds indicate point estimates; vertical bars indicate 95% CIs.

The 4-month cumulative seroincidence at baseline was 42.1% (95% CI 36.6%–47.6%) in RS, 45.8% (95% CI 37.1%−54.4%) in RT, and 36.2% (95% CI 30.3%–42.1%) in MT. We saw a strong control effect in all 3 approaches with statistically significant (p<0.001) reduction in seroincidence from baseline to study end. In RS, the relative decrease was 66.4% and the absolute decrease was 28.0 (95% CI 22.5–33.4) percentage points. In RT, the relative decrease was 69.4% and the absolute decrease was 31.8 (95% CI 20.1–43.4) percentage points. In MT, the relative decrease was 64.9% and the absolute decrease was 23.5 (95% CI 15.2–31.7) percentage points (Figure 4). The most rapid decrease occurred with RS, in which maximum effect was reached after 8 months, and remained stable thereafter. We did not see a significant difference in reduction of seroincidence between any 2 pairs of study approaches during the 24 month-study (RT vs. MT, p = 0.27; RT vs. RS, p = 0.55; RS vs. MT, p = 0.40).

### Prevalence of Taeniasis

At study end, 81.7% (7,248/8,873) of age-eligible persons accepted treatment for taeniasis; 6,537 (73.6%) provided a posttreatment stool sample. The unadjusted prevalence of taeniasis was 0.72% (17/2,349) in RS, 1.31% (29/2,206) in RT, and 0.40% (8/1,977) in MT. After adjusting for number of pigs in the village, baseline village seroprevalence, participant age, and the clustered study design, the model-estimated prevalence of taeniasis was 0.74% (95% CI 0.14%–3.81%) in RS, 1.09% (95% CI 0.21%–5.61%) in RT, and 0.62% (95% CI 0.11%–3.46%) in MT (Table 5). In villages that received a targeted strategy, most (78.2%; 36/46) persons who had taeniasis at study end lived in households that were not identified for intervention by using the ring approach.

**Table 5:** Taeniasis frequency and prevalence by study arm after 24 months of *Taenia solium* intervention, Peru

Study arm	No. taeniasis cases	No. stool samples tested	Prevalence, %	
			Crude	Adjusted* (95% CI)
Ring screening				
Pig treatment	3	1,155	0.26	0.32 (0.07–1.45)
No pig treatment	14	1,194	1.17	0.89 (0.22–3.56)
Ring treatment				
Pig treatment	14	1,107	1.26	0.55 (0.09–3.23)
No pig treatment	15	1,099	1.36	1.54 (0.37–6.51)
Mass treatment				
Pig treatment	4	992	0.40	0.69 (0.16–2.86)
No pig treatment	4	985	0.41	0.46 (0.09–2.33)

*Adjusted for number of pigs in the village, baseline village seroprevalence, participant age, and the clustered study design.

### Antiparasitic Treatment for Pigs

**Figure 5:**
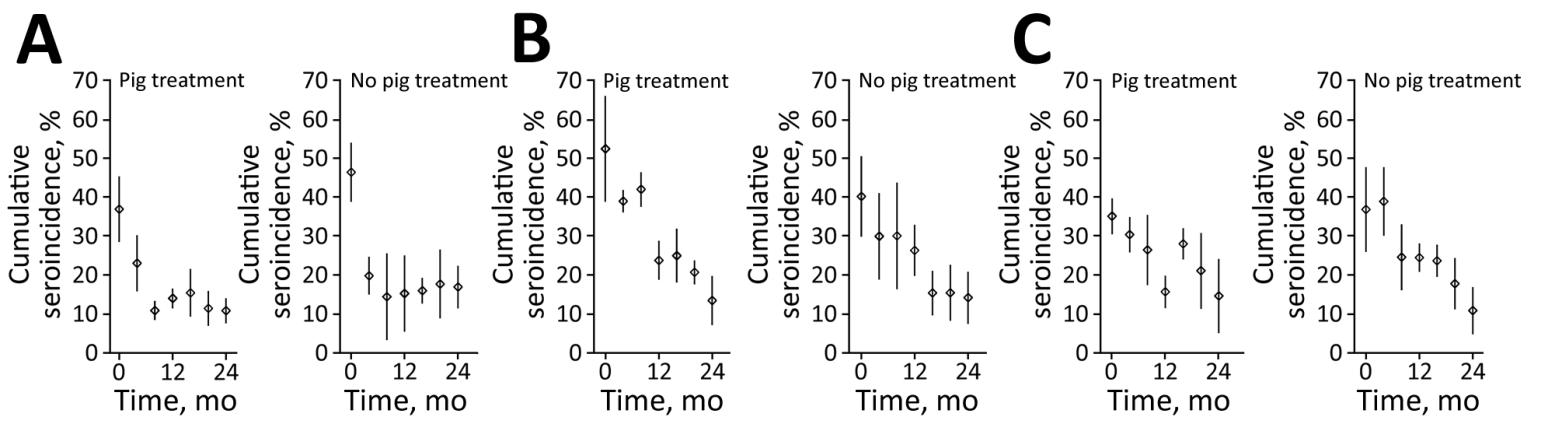
Comparison of cumulative *Taenia solium* seroincidence among pigs by study arm over time, Peru. A) Ring screening; B) ring treatment; C) mass treatment. Each intervention approach used niclosamide for human taeniasis in villages. Each approach included 2 arms: 1 with oxfendazole treatment of pigs for cysticercosis and 1 without pig treatment. In ring screening, participants living near pigs with cysticercosis were screened for taeniasis using stool coproantigen; identified cases were treated with niclosamide. In ring treatment, participants living near pigs with cysticercosis received presumptive treatment with niclosamide. In mass treatment, participants received treatment with niclosamide every 6 months regardless of location. Diamonds indicate point estimates; vertical bars indicate 95% CIs.

Adding oxfendazole treatment for pigs did not provide additional benefit and did not decrease overall pig seroincidence in any of the 3 approaches (Figure 5). We saw no statistically significant interaction between study arm and oxfendazole treatment; treatment was not a statistically significant covariate in the full model. The model-estimated seroincidence was 20.9% (95% CI 19.0%– 22.8%) in nontreated pigs compared with 21.9% (95% CI 20.2%–23.7%) in treated pigs.

## Discussion

We found that targeted delivery of niclosamide to treat and prevent human taeniasis in a ring strategy and uniform delivery in MDA both effectively reduced *T. solium* transmission. All 3 tested intervention approaches achieved >65% reduction in porcine *T. solium* seroincidence during the 2-year study, and all 3 were accepted broadly within study communities. 

Ideal control approaches for taeniasis and cysticercosis might vary across regions, and such approaches should consider which resources and infrastructure are available locally. Niclosamide MDA might be the easiest strategy to implement because of the extensive worldwide experience with this approach for other neglected tropical diseases. Primary benefits of MDA include operational simplicity and familiarity. In our study, *T. solium* transmission decreased steadily over time during repeated rounds of niclosamide at 6-month intervals. Niclosamide is safe for the general population (*8*) because it does not provoke brain inflammation in persons with neurocysticercosis, which is a concern in using the alternative drug, praziquantel (*26*). On the other hand, MDA is particularly inefficient for treating taeniasis. Unlike other neglected tropical diseases for which MDA is used, endemic *T. solium* transmission is sustained by a low prevalence of taeniasis, typically 1%–3%. Therefore, MDA for taeniasis applies most drugs to persons who are not infected and who might have limited risk for disease. Other drawbacks of MDA include more of the population exposed to possible adverse events, declining participation over time, and mixed evidence of sustained effect of MDA on transmission (*27*).

Ring strategy is applied on the premise that targeting high-risk subpopulations with niclosamide can achieve taeniasis control by treating fewer persons than in MDA, which ignores known spatial risk heterogeneity (*17*). Although only 19.3% of our study population received niclosamide through RT whereas 88.5% of persons received it through MDA, we saw no difference in reduction of transmission between the 2 approaches. The main disadvantage of ring strategy is operational complexity; this strategy requires surveillance to detect heavily infected pigs and identify focal areas for intervention. We used centralized active surveillance in which dedicated veterinary teams screened the pig population every 4 months. This approach might be difficult to implement on a large scale, particularly in impoverished rural regions isolated from government resources and attention.

For programmatic application of ring strategy, passive community surveillance with incentives for reporting could be more pragmatic. In this strategy, residents would report meat visibly contaminated with cysts at time of slaughter or animals found to be tongue-positive during sale, thus prompting RT with niclosamide by community health workers. We pilot tested this approach in Peru and found that passive surveillance without incentives did not achieve sufficient reports and drug delivery to reduce parasite transmission (*28*). Pigs provide cash income to villagers who sell their animals to offset unanticipated economic needs. Loss of income at these crucial moments was a strong disincentive to report and often resulted in consuming or selling contaminated meat. However, in another pilot study in the same region, strong community engagement with incentives resulted in sufficient reporting to control transmission (S. O’Neal, unpub. data). We are conducting implementation research for programmatic application of RT in Peru.

Screening for taeniasis followed by treatment for diagnosed cases is an alternative to presumptive treatment. Mass stool screening is infeasible on a large scale because of cost and operational complexity, but ring strategy enables targeted application of screening resources. In our study, screening reduced the proportion of the population receiving niclosamide to 1.4% in RS versus 19.3% in RT while maintaining control effectiveness but did so at additional cost and complexity due to collection and processing of stool samples. A screening approach for taeniasis using the most sensitive test, coproantigen ELISA, might not be possible in regions without laboratory infrastructure or access to reagents, which remains a barrier to screening in most endemic areas (*29*).

In regions with robust veterinary infrastructure, control interventions in the pig population, such as treatment with oxfendazole or immunization with highly effective vaccines (*13*), could be applied as a standalone program or in combination with treatment for taeniasis. All the strategies we tested had treatment for taeniasis as the core intervention because taeniasis is the most prolific *T. solium* life stage and direct cause of cysticercosis in humans and pigs. Of note, we saw no additional reduction in transmission in any study approach when we added oxfendazole treatment for pigs. This finding suggests that when sustained control pressure is applied to humans as the definitive host, additional interventions in the intermediate pig host might not be necessary. We did not test oxfendazole in the absence of treatment for taeniasis; therefore, we cannot draw conclusions on the effectiveness of treatment interventions exclusively in pig versus human populations. We also did not apply vaccines against porcine cysticercosis, but this option could be considered in both mass and targeted approaches (*30*).

The strengths of our study were cluster-randomized design, head-to-head evaluation of interventions, and 2-year duration of the intervention. Limitations include that the small number of clusters in each study arm limited precision of outcome estimates, which could have affected our ability to distinguish true differences between arms. However, results and interpretations were consistent using multiple methods for determining SEs with small numbers of clusters, and we reported results using the most conservative method. The factorial design and large number of pigs in each cluster also benefited study efficiency. We randomly assigned villages to interventions, but the groups differed with respect to the proportion of households with pig corrals and latrines and the baseline seroprevalence of porcine cysticercosis. We controlled for these factors in the analysis, but residual confounding or differences in other unmeasured risk factors might have contributed to observed differences in outcomes. Participation in the studied interventions likely would differ across regions and cultures. In addition, ring interventions likely are dependent on geographic features, such as terrain and housing density. Thus, the results of this study might not be the same in regions where these factors differ. Finally, the secondary outcome measure of taeniasis prevalence at study end should be interpreted with caution because a baseline measurement was not taken. Diagnosis of taeniasis obligates treatment, so baseline measurement of taeniasis was not done because it would have confounded the interventions under evaluation.

In conclusion, our findings clearly demonstrate that substantial and rapid *T. solium* control can be achieved by using existing technology. Government control programs for taeniasis and cysticercosis can be initiated and scaled in accordance with the World Health Organization roadmap for overcoming neglected tropical diseases (*7*).

Appendix Additional information on targeted and mass drug administration interventions to control *Taenia solium*, Peru.
