# Supplementary material for: Geographically Targeted Interventions versus Mass Drug Administration to Control Taenia solium Cysticercosis, Peru
Source: Emerg Infect Dis. 2021 Sep;27(9):2389–98. doi: 10.3201/eid2709.203349 (PMC8386790; doi:10.3201/eid2709.203349)
Supplement: Appendix — Additional information on targeted and mass drug administration interventions to control Taenia solium, Peru. [file 20-3349-Techapp-s1.pdf]

Article DOI: https://doi.org/10.3201/eid2709.203349

Geographically Targeted Interventions versus Mass Drug Administration to Control *Taenia solium* Cysticercosis, Peru

Appendix

Additional Methods

Sample Size Calculations

We used a 2-sample proportion test in Stata SE12.1 (StataCorp LLC, https://www.stata.com) to calculate sample size requirements for the primary outcome, cumulative seroincidence among pigs, between intervention groups. Assuming a 60% reduction in cumulative incidence from baseline to end in the reference group, ≈800 pigs were needed per group to detect a between-group difference ≥7 percentage points at a power of 0.8 (2-sided $\alpha = 0.05$). We allocated villages on the basis of a conservative estimate of the ratio of the human–pig population, to meet the sample size requirements.

Intervention Group Allocation

Among possible villages, 23 met the criteria for population size of 50–500 residents, accessibility by vehicle, and no history of control interventions for taeniasis or cysticercosis. We used Stata SE14.0 (StataCorp, LLC) to allocate each of the 23 villages randomly into 1 of 6 intervention groups. We used the *generate rannum = uniform()* command, with a specified seed, to assign a random number to each village from a uniform distribution. The villages were sorted numerically in ascending order by their random number, then sequentially allocated to each intervention in blocks of 4 villages; the last block included only 3 villages. We repeated this sequence until the number of residents in each intervention group was within 10% of the total study population divided by 6; groups contained 1,415–1,731 residents each, per a prestudy census of 9,438 total residents in all 23 villages. No other factors were considered in assigning villages to interventions.

Consent Procedures

Prior to the start of the study, we met with leaders in each community to gauge their interest in participating and to request permission to include the village in the study. We also held open community meetings to provide information on the objectives, study design, procedures, anticipated start date, and to solicit comments. At the start of the study, our teams visited each home in the selected villages to provide additional information both verbally and in writing, to answer questions, and to solicit informed consent. During our subsequent visits to the villages to provide study interventions, we also recruited newly arrived residents for enrollment and participation in the study. Adults who chose to participate were required to sign a written consent form with complete information about the study, their rights as participants, and the contact information for study personnel and for members of the oversight board for the study. A parent or legal guardian was required to authorize the participation of any child 2–17 years and complete and sign a separate consent form for this purpose.

Collection and Processing of Pig Serum

Veterinary study teams collected blood samples from pigs by going door-to-door in study villages. We maintained a continuous cold chain from the point of collection all the way through laboratory processing. Blood samples were collected by venipuncture of the anterior vena cava and were placed immediately on ice in coolers in the villages. Samples were transferred several times each day to a temporary field laboratory, where they were centrifuged and 1.5 mL aliquots of serum were placed in microtubules and stored at −20°C. Frozen serum samples then were shipped by air to the Parasite Immunodiagnostics Laboratory at the Universidad Peruana Cayetano Heredia (Lima, Peru), where they were analyzed by lentil-lectin glycoprotein enzyme-linked immunoelectrotransfer blot (LLGP EITB) for presence of antibodies against *Taenia solium* cysts, as previously described (*1*,*2*). As an antibody detection test, LLGP EITB does not distinguish exposure from infection, nor active infection from cleared infection. Nonetheless, LLGP EITB indicates pig exposure to *T. solium* oncospheres; thus, population-level results can be used to monitor transmission of *T. solium* in a pig population.

LLGP EITB is based on a semi-purified fraction of 7 native *T. solium* glycoprotein (GP) antigens (GP50, GP42–39, GP24, GP21, GP18, GP14, and GP13) and the antigen number indicates the molecular weight in kDa. The assay was originally reported to be 100% sensitive and 100% specific in well-defined sets of serum, but only a limited number of potentially cross-

reactive parasite species were evaluated (*2*). We excluded GP50 in this study because recent studies showed that this band cross-reacts with *T. hydatigena*, a cestode that infects pigs and is co-endemic in the region where we conducted our study (*3*,*4*). We anticipated this change would reduce the sensitivity of the assay because GP50 is typically the first reactive band to appear after exposure, but we also expected the change to increase the assay's specificity. Because the GP50 band was excluded equally from the results in all study arms, we do not expect this change to affect interpretation of comparative results.

Collection and Processing of Human Stool

Our study teams went door-to-door in all study villages to offer presumptive treatment with niclosamide (NSM) for taeniasis, and to provide each participant with a 500-mL plastic container, soap, and instructions for hygienic collection of a posttreatment whole-stool sample. We collected posttreatment stool samples to increase the likelihood of detecting parasite material. We returned to each home within 24 hours to collect stool samples from participants and transferred these immediately to a temporary field laboratory. There, we examined samples macroscopically for the presence of *Taenia* sp. scoleces or proglottids, then placed 10-mL fecal aliquots in 40 mL of 5% formol-phosphate buffered saline at pH 7.2 in sealed propylene tubes. These samples were transported by ground at ambient temperature to the Center for Global Health laboratory (Tumbes, Peru) where they were concentrated by spontaneous sedimentation for 24 hours, then examined by light microscopy for the presence of *Taenia* sp. eggs (*5*). We then shipped the fecal samples by air to the CNS Parasitic Diseases Research Unit, Universidad Peruana Cayetano Heredia (Lima, Peru) for further analysis by ELISA to detect *Taenia* sp. coproantigens, as previously described (*6*), with the exception that the capture antibody and conjugate used were specific to *T. solium* (*7*). This ELISA assay has 96.4% sensitivity and 100% specificity for *T. solium* (*7*). We used posttreatment stool samples and multiple evaluation techniques (macroscopy, microscopy, ELISA) to maximize the likelihood of detecting true positive cases.

References

1. Tsang VC, Brand JA, Boyer AE. An enzyme-linked immunoelectrotransfer blot assay and glycoprotein antigens for diagnosing human cysticercosis (*Taenia solium*). J Infect Dis. 1989;159:50–9. PubMed https://doi.org/10.1093/infdis/159.1.50

2. Tsang VCW, Pilcher JA, Zhou W, Boyer AE, Kamango-Sollo EIO, Rhoads ML, et al. Efficacy of the immunoblot assay for cysticercosis in pigs and modulated expression of distinct IgM/IgG activities to *Taenia solium* antigens in experimental infections. Vet Immunol Immunopathol. 1991;29:69–78. PubMed https://doi.org/10.1016/0165-2427(91)90053-F

3. Muro C, Gomez-Puerta LA, Flecker RH, Gamboa R, Barreto PV, Dorny P, et al.; for the Cysticercosis Working Group In Peru. Porcine cysticercosis: possible cross-reactivity of *Taenia hydatigena* to GP50 antigen in the enzyme-linked immunoelectrotransfer blot assay. Am J Trop Med Hyg. 2017;97:1830–2. PubMed https://doi.org/10.4269/ajtmh.17-0378

4. Gomez-Puerta L, Vargas-Calla A, Castillo Y, Lopez-Urbina MT, Dorny P, Garcia HH, et al.; Cysticercosis Working Group in Peru. Evaluation of cross-reactivity to *Taenia hydatigena* and *Echinococcus granulosus* in the enzyme-linked immunoelectrotransfer blot assay for the diagnosis of porcine cysticercosis. Parasit Vectors. 2019;12:57. PubMed https://doi.org/10.1186/s13071-018-3279-5

5. Tello R, Terashima A, Marcos LA, Machicado J, Canales M, Gotuzzo E. Highly effective and inexpensive parasitological technique for diagnosis of intestinal parasites in developing countries: spontaneous sedimentation technique in tube. Int J Infect Dis. 2012;16:e414–6. PubMed https://doi.org/10.1016/j.ijid.2011.12.017

6. Allan JC, Velasquez-Tohom M, Torres-Alvarez R, Yurrita P, Garcia-Noval J. Field trial of the coproantigen-based diagnosis of *Taenia solium* taeniasis by enzyme-linked immunosorbent assay. Am J Trop Med Hyg. 1996;54:352–6. PubMed https://doi.org/10.4269/ajtmh.1996.54.352

7. Guezala M-C, Rodriguez S, Zamora H, Garcia HH, Gonzalez AE, Tembo A, et al. Development of a species-specific coproantigen ELISA for human *Taenia solium* taeniasis. Am J Trop Med Hyg. 2009;81:433–7. PubMed
